# Endobronchial Ultrasound Transbronchial Needle Aspiration in Thoracic Diseases: Much More than Mediastinal Staging

**DOI:** 10.1155/2018/4269798

**Published:** 2018-03-04

**Authors:** Juliana Guarize, Monica Casiraghi, Stefano Donghi, Cristina Diotti, Nicolo Vanoni, Rosalia Romano, Chiara Casadio, Daniela Brambilla, Patrick Maisonneuve, Francesco Petrella, Lorenzo Spaggiari

**Affiliations:** ^1^Division of Thoracic Surgery, European Institute of Oncology, Milan, Italy; ^2^Division of Pathology, European Institute of Oncology, Milan, Italy; ^3^Division of Epidemiology and Biostatistics, European Institute of Oncology, Milan, Italy; ^4^Department of Oncology and Hemato-Oncology, University of Milan, Milan, Italy

## Abstract

**Background and Objective:**

EBUS-TBNA has revolutionized the diagnostic approach to thoracic diseases from a surgical to minimally invasive procedure. In non small-cell lung cancer (NCSLC) patients, EBUS-TBNA is able to dictate the consecutive therapy both for early and advanced stages, providing pathological diagnosis, mediastinal staging, and even adequate specimens for molecular analysis. This study reports on the ability of EBUS-TBNA to make different diagnoses and dictates the consecutive therapy in a large cohort of patients presenting different thoracic diseases.

**Methods:**

All procedures performed from January 2012 to September 2016 were reviewed. Five groups of patients were created according to the main indications for the procedure. Group 1: lung cancer staging; Group 2: pathological diagnosis in advanced stage lung cancer; Group 3: lymphadenopathy in previous malignancies; Group 4: pulmonary lesions; Group 5: unknown origin lymphadenopathy. In each group, the diagnostic yield of the procedure was analysed. Non malignant diagnosis at EBUS-TBNA was confirmed by a surgical procedure or clinical and radiological follow-up.

**Results:**

1891 patients were included in the analysis. Sensitivity, negative predictive value, and diagnostic accuracy in each group were 90.7%, 79.4%, and 93.1% in Group 1; 98.5%, 50%, and 98.5% in Group 2; 92.4%, 85.1%, and 94.7% in Group 3; 90.9%, 51.0%, and 91.7% in Group 4; and 25%, 83.3%, and 84.2% in Group 5. Overall sensitivity, negative predictive value, and accuracy were 91.7%, 78.5%, and 93.6%, respectively.

**Conclusions:**

EBUS-TBNA is the best approach for invasive mediastinal investigation, confirming its strategic role and high accuracy in thoracic oncology.

## 1. Introduction

Mediastinal adenopathy has always been assessed by radiological imaging such as computed tomography (CT) and positron emission tomography (PET), with high sensitivity but a low diagnostic accuracy for the purposes of correct clinical decision-making [1]. To date, mediastinoscopy has been considered the gold standard for diagnosis and mediastinal staging with high sensitivity and accuracy, but the procedure has been progressively underused due to its high invasiveness, risk of complications, and the need to be performed in experienced centres [2].

In the early 2000's, a minimally invasive convex probe endobronchial ultrasound (EBUS) procedure able to perform real-time transbronchial needle aspiration (TBNA) was described with high accuracy for mediastinal and hilar lymph node staging [3]. Since then, EBUS-TBNA has gradually changed the way mediastinal staging is performed and rapidly improved its value with new indications in lung cancer management, becoming the standard of care [4].

In thoracic oncology, EBUS-TBNA has revolutionized the diagnostic approach to lung cancer and other neoplasms from a surgical to minimally invasive procedure. Especially in non small-cell lung cancer (NCSLC), EBUS-TBNA is able to dictate the consecutive therapy both for early and advanced stages, providing pathological diagnosis, mediastinal staging, and even adequate specimens for molecular analysis [5]. In addition, EBUS-TBNA has been described in different clinical scenarios, particularly for the diagnosis and definition of granulomatosis such as sarcoidosis [6] and tuberculosis [7] and for pathological assessment of mediastinal and hilar recurrences from previous malignancies [8, 9].

This study reports the largest published experience in the use of EBUS-TBNA in a high-volume thoracic oncology institution. We aimed to describe the utility and diagnostic yield of EBUS-TBNA in different clinical scenarios in thoracic diseases, dividing our series into five different groups according to the main indication for the procedure (Group 1: lung cancer staging, Group 2: pathological diagnosis in advanced stage lung cancer, Group 3: lymphadenopathy in previous malignancies, Group 4: pulmonary lesions, and Group 5: unknown origin lymphadenopathy) and reporting our results in terms of sensitivity, negative predictive value, and diagnostic accuracy.

## 2. Methods

This single-centre retrospective study with a prospective follow-up was approved by the Institutional Review Board and the individual consent was obtained.

From January 2012 to September 2016, 1958 EBUS-TBNA procedures were performed at our institution. The indications for EBUS-TBNA, lymph node stations, number of lymph nodes sampled, cytological results, and cancer cell type were obtained for the analysis. To better standardize the series, different groups of patients were defined according to the indication for the procedure.

Group 1 included patients referred for EBUS-TBNA for mediastinal staging in potentially operable lung cancer and patients with mediastinal involvement but no evidence of distant metastasis. Patients with proven or suspected NSCLC were included, and pathological cell type was performed in the same procedure. According to our institutional protocol, suspect lymph nodes were defined as lymph nodes with a pathological PET scan uptake and/or enlarged lymph nodes with more than 1 cm in the short axis at the CT scan.

Group 2 included patients with metastatic and/or bulky mediastinal disease referred for EBUS-TBNA for pathological diagnosis and molecular mutational analysis for targeted therapy; Group 3 included patients with a previous (thoracic or extrathoracic) malignancy who developed mediastinal and hilar lymphadenopathies suspected for recurrence; Group 4 included patients who underwent EBUS-TBNA for primary tissue sampling in pulmonary lesions (paratracheal or peribronchial); and Group 5 included patients with mediastinal and hilar lymphadenopathy of unknown origin with no history of malignancy. Diagnostic sensitivity, accuracy, and negative predictive value were calculated according to standard definitions. Sensitivity was calculated for the diagnosis of malignancy.

EBUS-TBNA samples were considered diagnostic when a definitive diagnosis was obtained. Lymph node samples negative for malignancy underwent surgical confirmation (mediastinoscopy or VATS) or clinical and radiological follow-up (at least 12 months of follow-up). Patients with loss of follow-up were excluded.

EBUS-TBNA samples were considered false negative when surgery changed the final diagnosis, or there was clinical and radiological progression of the disease during follow-up. EBUS-TBNA samples insufficient or inadequate for diagnosis were considered false negative in the calculation of diagnostic accuracy. Patients without a specific diagnosis at EBUS-TBNA (benign or malignant) but a definitive non-malignant diagnosis at mediastinoscopy were considered false negative.

During EBUS-TBNA procedures, all lymphadenopathies with increased PET scan pathological uptake were sampled in patients with a non-malignant diagnosis at rapid on-site evaluation (ROSE), and at least one mediastinal and two hilar stations were sampled when ROSE was negative for tumour cells and suspected for lymphadenitis granulomatosis.

Patients with negative EBUS-TBNA samples were referred for confirmatory surgical procedures after a multidisciplinary team discussion with thoracic surgeons, pulmonologists, radiologists, oncologists, and radiotherapists when lymphadenopathies were considered highly suspicious for recurrence based on CT and/or PET scan characteristics.

### 2.1. EBUS-TBNA Technical Aspects

EBUS-TBNA procedures were performed under local anaesthesia (1% lidocaine), and moderate sedation was provided by an anaesthesiologist with spontaneous ventilation. The same team of interventional pulmonologists using a convex probe (EBUS Convex Probe BF-UC180F; Olympus) and a dedicated ultrasound processor (EU-ME1; Olympus) performed all procedures. All EBUS-TBNA specimens were collected with a 22 gauge dedicated needle (Vizishot NA-201SX-4022; Olympus).

A very small amount of the aspirated material was pushed out by the internal stylet and smeared onto glass slides for immediate on-site evaluation (ROSE). Mirror slides were alcohol-fixed for posterior evaluation. Air-dried smears were immediately stained with a modified May-Grünwald Giemsa stain (MGG Quick Stain; Bio-Optica, Milan, Italy) and evaluated by the cytopathologist to confirm adequate tumour cells and/or lymph node material. The remaining aspirate and other needle passages (at least three needle passages each station) were fixed in a formalin solution for cell block processing and histological evaluation. Alcohol-fixed smears were stained with Papanicolaou and haematoxylin-eosin stains.

## 3. Results

Out of 1958 patients, 1891 were included in the study. Sixty-seven patients were excluded from statistical analysis due to loss of follow-up with the impossibility to confirm the negativity of lymph node samples. Patients' mean age was 65 years (range: 20–92), 1197 (63.3%) were men.

The leading indication for EBUS-TBNA at our institution was lung cancer staging in 728 (38.5%) patients followed by pathological diagnosis and mutational status in advanced stage lung cancer in 401 (21.2%) patients, lymphadenopathy in previous malignancies in 320 (17%) cases, diagnosis of pulmonary lesions in 290 (15.3%), and diagnosis of unknown origin lymphadenopathy in 152 (8%) patients. Patient characteristics and indications for the procedures are reported in Table 1.

A total of 2298 lymph node stations were sampled: 1003 superior mediastinal: 2 high upper station #1, 56 station #2R, 8 station #2L, 740 station #4R, and 197 station #4L; 789 subcarinal station #7; 506 N1 nodes: 57 hilar (37 station #10R and 20 station #10L), 365 interlobar (191 station #11R and 174 station #11L), 83 lobar (53 station #12R and 30 station #12L), and 1 segmental station #13L. Lymph node sampled locations and number of lymph nodes are listed in Table 2.

Cytological diagnoses were adenocarcinoma in 692 patients, squamous cell carcinoma in 216, neuroendocrine tumours in 143 (102 small-cell lung cancer (SCLC), 19 large-cell neuroendocrine tumours, 9 mixed large- and small-cell neuroendocrine tumours, 10 typical carcinoids, 3 atypical carcinoids), 154 metastases from extrathoracic malignancy, 65 poorly differentiated NSCLC, 24 lymphoproliferative disorders, 34 other malignancies (1 thymoma, 8 mesothelioma, 3 adenoid cystic carcinoma, 6 mixed tumours, 9 poorly differentiated epithelial tumours of unknown origin, 1 sarcoma, and 3 sarcomatoid carcinomas), 100 lymphadenitis granulomatosis (97 sarcoid-like reaction and 3 tuberculosis), 316 normal/reactive lymph nodes, and 28 other non-neoplastic diagnoses (Table 3).

Overall sensitivity, negative predictive value, and diagnostic accuracy were 91.7% (95% CI: 90.1–93.0), 78.5% (95% CI: 74.9–81.7), and 93.6% (95% CI: 92.4–94.6), respectively.

### 3.1. Group 1: Lung Cancer Staging

Out of 728 (60.2%) patients, 438 underwent EBUS-TBNA for mediastinal staging and the diagnosis of the cell-type tumour in the same procedure, and 244 (33.5%) had a previous pathological diagnosis of NSCLC, and EBUS-TBNA was performed for complete mediastinal staging, and in 46 (6.3%) patients, EBUS-TBNA was performed for restaging the mediastinum after induction chemotherapy for stage IIIA (pN2) NSCLC. In this group, EBUS-TBNA was diagnostic for malignancy in 485 (66.6%) patients, and a mean of 1, 5 lymph nodes per patient was biopsied. In 187 (25.7%) cases, EBUS-TBNA revealed normal or reactive lymph node samples, and in six (0.8%), EBUS-TBNA revealed a granulomatous reaction in five cases of sarcoid-like reaction compatible with sarcoidosis and one case of necrotizing granulomatosis with a positive culture for *Mycobacterium tuberculosis*.

Malignant diagnoses included 278 adenocarcinoma, 102 squamous cell carcinoma, 55 neuroendocrine, 27 poorly differentiated nonsmall-cell lung cancer, and 23 other malignancies (6 metastasis from a nonthoracic malignancy, 5 lymphoma, 4 mesothelioma, 1 carcinosarcoma, 1 mixed squamous cell and adenocarcinoma, and 6 undifferentiated epithelioid neoplasms).

Fourty-six patients underwent EBUS-TBNA for restaging the mediastinum after neoadjuvant chemotherapy. There were 16 out of 46 (34.8%) positive for malignancy and 6 (13%) false negative cases. From false negative cases, four patients received chemotherapy and mediastinal radiotherapy and two patients' neoadjuvant chemotherapy. Sensitivity, negative predictive value, and diagnostic accuracy in this subgroup of patients were 66.7% (95% CI: 45.4–82.8), 78.1% (95% CI: 61.2–89), and 84.8% (95% CI: 71.8–92.4), respectively.

Among 193 patients with a nonmalignant diagnosis at EBUS-TBNA, 146 underwent surgical procedures for primary tumours confirming the negativity of lymph node samples, and 47 patients were excluded from surgery for clinical reasons and underwent a mean of 17.9 month's clinical and radiological follow-up. In this group, there were 50 (6.9%) false-negative samples. Sensitivity, negative predictive value, and diagnostic accuracy in the staging group were 90.7% (95% CI: 87.9–92.8), 79.4% (95% CI: 73.9–84.0), and 93.1% (95% CI: 91.1–94.8), respectively.

### 3.2. Group 2: Pathological Diagnosis and Mutational Status in Advanced Stage Lung Cancer

This group comprised 401 patients. In 344 (85.8%) patients, EBUS-TBNA was performed for the first cell type diagnosis in stages IIIB and IV lung cancer, and in 57 (14.2%) patients, the procedure was performed after an initial established pathological diagnosis for tissue sampling for molecular analysis including the epidermal growth factor (EGFR) mutational profile, anaplastic lymphoma kinase (ALK) fusion genes, and mesenchymal-epithelial transition (MET) protooncogene amplification. Sensitivity, negative predictive value, and diagnostic accuracy in this group were 98.5% (95% CI: 96.7–99.3), 50% (95% CI: 25.4–74.6), and 98.5% (95% CI: 96.8–99.3), respectively.

### 3.3. Group 3: Lymphadenopathy in Previous Malignancy

Out of 320 (59.4%) patients, 190 had a diagnosis of a recurrence of a previous tumour and 113 (35.3%) had a different diagnosis from the previous tumour. There were 17 (5.3%) false negative cases. In 97 patients, EBUS-TBNA revealed a definitive nonmalignant diagnosis, and 35 patients had evidence of a granulomatosis disease. Twenty-two out of 114 (19.3%) patients with nonmalignant diagnoses at EBUS-TBNA underwent mediastinoscopy or other surgical procedures to confirm the negativity of EBUS-TBNA diagnosis, and 92 (80.7%) patients underwent clinical and radiological median follow-up of 17 months. Sensitivity, negative predictive value, and diagnostic accuracy were 92.4% (95% CI: 88.1–95.2), 85.1% (95% CI: 77.4–90.5), and 94.7% (95% CI: 91.7–96.7), respectively.

### 3.4. Group 4: Pulmonary Lesions

EBUS-TBNA was diagnostic for malignancy in 241 out of 290 (83.1%) cases. Cytological diagnoses were 10 lymphoproliferative disorders, 95 adenocarcinomas, 55 squamous cell carcinoma, 25 neuroendocrine tumours, 29 metastasis from other neoplasms, 14 poorly differentiated nonsmall-cell lung cancer, and 13 other malignancies (2 mixed adenocarcinomas and neuroendocrine, 2 adenoid cystic carcinomas, 1 pleomorphic carcinoma, 1 sarcomatoid carcinoma, 1 nonspecific CTM, 3 epithelioid mesotheliomas, 1 poorly differentiated epithelioid neoplasm, 1 mesenchymal neuron neoplasm, and 1 sarcoma). EBUS-TBNA revealed a nonmalignant diagnosis in 25 cases: 5 hamartomas, 4 fibrotic lesions after radiotherapy, 6 bronchogenic cyst, 1 pericardial cyst, 1 benign leiomyoma, 5 pneumonias, 1 mycetoma and 2 cases of granulomatosis (1sarcoidosis and 1 tuberculosis). In this group of patients, sensitivity, negative predictive value, and diagnostic accuracy were 90.9% (95% CI: 86.9–93.8), 51.0% (95% CI: 37.5–64.4), and 91.7% (95% CI: 88.0–94.4), respectively.

### 3.5. Group 5: Unknown Origin Lymphadenopathy

This group included 152 patients: 59 granulomatosis lymphadenitis, 60 reactive lymph nodes, 3 lymphoproliferative disorders, 2 adenocarcinomas, 2 metastasis from other sites (thyroid and breast), 1 small-cell lung cancer, 1 ectopic thymus tissue, and 24 false negative samples. Sensitivity, negative predicted value, and diagnostic accuracy were 25% (95% CI: 13.3–42.1), 83.3% (95% CI: 76.4–88.5), and 84.2% (95% CI: 77.6–89.2), respectively. The diagnostic performance of EBUS-TBNA in the different groups of patients according to clinical indication for the procedure is shown in Table 4.

## 4. Discussion

Many literature studies have reported on EBUS-TBNA experience for mediastinal staging in lung cancer patients and some have described the utility of EBUS-TBNA in other pathologies with mediastinal lymphadenopathies such as sarcoidosis, tuberculosis, and lymphoma [10, 11].

During the last decade, EBUS-TBNA has become an essential diagnostic procedure in thoracic disease, revolutionizing the approach to diagnosis and treatment and guiding the best treatment option in a large number of patients. To date, the procedure has been widely used to perform mediastinal staging and has been included in almost all guidelines as the preferred first approach for invasive mediastinal staging in lung cancer patients [4, 12].

A recent meta-analysis showed a sensitivity of EBUS-TBNA for mediastinal staging ranging from 81 to 95% [13]. Likewise, an Italian multicentre trial published in 2017 analysed 485 patients showing a sensitivity of 90% with a diagnostic accuracy of 93% [14]. In agreement with these publications, our study showed an EBUS-TBNA sensitivity for malignant disease of 90.7% and a diagnostic accuracy of 93.1% in 728 patients in our Group 1 patients (lung cancer staging). Surprisingly, the final EBUS-TBNA diagnosis was lymphoma in five patients with a single pulmonary lesion and mediastinal lymphadenopathies highly suspected for lung cancer. EBUS-TBNA samples were able to subtype the lymphoma in three of the five (60%) cases achieving a definitive diagnosis of Hodgkin's lymphoma, classic variation, showing the feasibility of lymphoma diagnosis and subclassification by EBUS-TBNA samples even if the need for further histological evaluation remains essential in many cases [15, 16]. In the subgroup of patients who underwent EBUS-TBNA for restaging the mediastinum after neoadjuvant chemotherapy, there were a relative higher incidence of inadequate samples due to the presence of fibrosis in the mediastinum and the difficulty of the procedure specially after chemoradiotherapy.

Considering our Group 2 (pathological diagnosis and mutational status in advanced stage lung cancer), EBUS-TBNA sensitivity and diagnostic accuracy were both 98.5%. EBUS-TBNA also provided adequate material for molecular analysis whenever requested in advanced stage adenocarcinomas in 98% of cases. This datum confirms our previous published experience of molecular analysis with EBUS-TBNA specimens where we showed a sensitivity of 96.9% [17]. Our previous study also demonstrated that molecular analysis obtained from EBUS-TBNA specimens was equivalent to that obtained from surgical specimens, with no differences in terms of sensitivity and diagnostic accuracy [17].

Due to the low invasive and the feasibility of repeated procedures, EBUS-TBNA is the ideal procedure to establish a cell-type diagnosis and to provide adequate specimens for molecular assay in advanced lung cancer patients. The procedure's high accuracy and low risk of complications make it suitable for low performance status patients who could still benefit from targeted therapy [18].

In Group 3 (lymphadenopathy in previous malignancy), EBUS-TBNA showed a sensitivity and diagnostic accuracy of 92.4% and 94.7%, respectively, in agreement with a previous similar study published by Navani et al. and also with our previous reported experience in this subset of patients [8, 9]. In this group of patients, EBUS-TBNA was a crucial diagnostic approach. The results of this study showed that 35.3% of patients with lymphadenopathy suspected for a recurrence did not really have a recurrence, and EBUS-TBNA revealed a different diagnosis. From all nonmalignant diagnoses, lymphadenitis granulomatosis was found in 36% of patients. EBUS-TBNA specimens provided adequate diagnosis avoiding unnecessary treatments in cases of nonmalignant disease and guiding therapy in cases of recurrence.

In the diagnosis of Group 4 patients (pulmonary lesions), EBUS-TBNA demonstrated a sensitivity and accuracy of 90.9% and 91.7%, respectively, in line with Nakajima et al.'s published data (sensitivity of 94.1% and diagnostic accuracy of 94.3% in the diagnosis of peribronchial and peritracheal lesions) [19]. Clinical presentations vary widely in this group, and EBUS-TBNA was able to establish a correct diagnosis in the vast majority of cases also in nonmalignant intrapulmonary lesions. Some interesting findings in this group included the diagnosis of a benign leiomyoma, a mycetoma, and ectopic thymus tissue.

In the investigation of Group 5 (unknown origin lymphadenopathy), EBUS-TBNA presented a diagnostic accuracy of 84.2%. Due to the very low prevalence of malignancy in this group of patients, sensitivity was not surprisingly very low (25%). The vast majority of patients in this group had an inflammatory disease with the most frequent diagnosis of granulomatous lymphadenitis compatible with sarcoidosis.

Our results refer to the largest published series in EBUS-TBNA, in a high-volume thoracic oncology institution, demonstrating the utility and high accuracy of EBUS-TBNA in different clinical scenarios in daily clinical practice.

Some additional findings have also emerged from this study. First, our study showed a wide use of EBUS-TBNA in elderly patients (732 patients over 70 years old and 120 over 80 years old), with a very low rate (0.74%) of postprocedure minor complications and no major events. Other interesting findings included the possibility to repeat biopsies easily and safely, obtaining tumour specimens for genetic assessment in stage IV NSCLC patients and in other tumour recurrences without the need for further more invasive procedures. These aspects highlight the importance of EBUS-TBNA as a diagnostic approach in different thoracic oncology scenarios, underpinning modern oncological strategy also for fragile patients and thereby avoiding more invasive and demanding procedures.

An essential aspect to achieve optimal results is the specimen handling in EBUS-TBNA [5]. The use of ROSE and synergy with the pathologist is crucial during the procedure both to obtain the best specimen collection and to achieve all pathological, immunohistochemistry, and molecular analyses. Despite some different previous points of view regarding ROSE [20–22], a recent metaexpert panel review showed that ROSE is necessary to reach all molecular analyses and to prevent invasive surgical procedures after EBUS-TBNA [23].

Our study has some limitations. The first is its retrospective nature, which probably limits and influences all of the variables included in the analysis. Another bias is that not all patients underwent a surgical procedure (mediastinoscopy or VATS) to confirm negative EBUS-TBNA samples but all patients had a clinical and radiological follow-up longer than 15 months.

In our experience, EBUS-TBNA is not just a technique, but a philosophy for the investigation of thoracic disease. EBUS-TBNA feasibility and optimal results largely depend on the cytopathology and pulmonologists' experience but also the way the procedure is performed. Adequate sedation and ROSE are crucial and should always be used. In experienced hands, EBUS-TBNA offers everything chest physicians need for the best medical practice without further invasive procedures. Surgical procedures are still required in highly suspicious NSCLC cases when EBUS-TBNA is negative for malignancy and in some cases for lymphoma subtyping.

In conclusion, EBUS-TBNA represents the best diagnostic approach in many different clinical scenarios in high-volume thoracic oncology centres and dictates the consecutive treatment option in the vast majority of patients. EBUS-TBNA is useful and accurate in a high percentage of cases underpinning modern oncological therapy, guiding the best treatment options in lung cancer and avoiding useless and invasive procedures in case of benign disease.

Based on our previous expertise, we are developing new applications for EBUS-TBNA such as a new protocol for EBUS-TBNA primary lung cancer cell culture and microRNA profile in stage IIIA (pN2) NSCLC patients, testing the feasibility of a microRNA profile obtained from EBUS-TBNA [24] and the correlation with the prediction of chemotherapy response. In addition, new molecular analyses are being developed, and the correlation between PDL-1 expression and tumour histology is under evaluation.

## Figures and Tables

**Table 1:** Patient characteristics and indications for EBUS-TBNA.

Variable	*N*° or median (range)
Patients (total)	1891
Sex	
Male	1197
Female	694
Age (years)	65 (20–92)
Indications for EBUS-TBNA	
Lung cancer staging	728
Pathological diagnosis and mutational status in advanced stage lung cancer	401
Lymphadenopathy in previous malignancy	320
Diagnosis of pulmonary lesions	290
Unknown origin lymphadenopathy	152

**Table 2:** Lymph node stations sampled by EBUS-TBNA in 1569 patients^∗^.

Stations	Right	Left
*Total*	**2298**	
*Superior mediastinal*	**1003**	
Highest mediastinal (station 1)	1	1
Upper paratracheal (2R/2L)	56	8
Lower paratracheal (4R/4L)	740	197
*Inferior mediastinal*		
Subcarinal (7)	789	
*N*1 *nodes*	**506**	
Hilar (10R/10L)	37	20
Interlobar (11R/11L)	191	174
Lobar (12R/12L)	53	30
Segmental (13R/13L)	0	1

^∗^Tissue sampling of pulmonary lesions in 322 cases.

**Table 3:** Cytological results of EBUS-TBNA.

Cytological results	Number of patients
Adenocarcinoma	692
Squamous cell carcinoma	216
Neuroendocrine tumour	143
SCLC	102
Large-cell neuroendocrine tumour	19
Mixed large- and small-cell neuroendocrine	9
Typical carcinoid	10
Atypical carcinoid	3
Metastasis from extrathoracic malignancy	154
NSCLC poorly differentiated (NAS)	65
Lymphoproliferative disorders	24
Other nonneoplastic diagnosis	28
Lymphadenitis granulomatosis	100
Sarcoid-like granulomas	97
Tuberculosis	3
Other malignancies^∗^	34
Reactive/normal lymph nodes	316

^∗^Other malignancies included 1 thymoma, 8 mesothelioma, 3 adenoid cystic carcinoma, 6 mixed tumours, 9 poorly differentiated epithelial tumours of unknown origin, 1 sarcoma, and 3 sarcomatoid carcinoma.

**Table 4:** Diagnostic performance of EBUS in the different groups.

EBUS	Overall	Group 1	Group 2	Group 3	Group 4	Group 5
Total	1891	728	401	320	290	152
True negative	441	193	6	97	25	120
False negative	121	50	6	17	24	24
True positive	1329	485	389	206	241	8
*Performance*	% (95% CI)	% (95% CI)	% (95% CI)	% (95% CI)	% (95% CI)	% (95% CI)
Sensitivity	91.7 (90.1–93.0)	90.7 (87.9–92.8)	98.5 (96.7–99.3)	92.4 (88.1–95.2)	90.9 (86.9–93.8)	25.0 (13.3–42.1)
Specificity	100 (99.1–100)	100 (98.0–100)	100 (61.0–100)	100 (96.2–100)	100 (86.7–100)	100 (96.9–100)
PPV	100 (99.7–100)	100 (99.2–100)	100 (99.0–100.)	100 (98.2–100)	100 (98.4–100)	100 (67.6–100)
NPV	78.5 (74.9–81.7)	79.4 (73.9–84.0)	50.0 (25.4–74.6)	85.1 (77.4–90.5)	51.0 (37.5–64.4)	83.3 (76.4–88.5)
Accuracy	93.6 (92.4–94.6)	93.1 (91.1–94.8)	98.5 (96.8–99.3)	94.7 (91.7–96.7)	91.7 (88.0–94.4)	84.2 (77.6–89.2)

## Abbreviations

EBUS-TBNA: Endobronchial ultrasound transbronchial needle aspiration
NSCLC: Nonsmall-cell lung cancer
ROSE: Rapid on-site evaluation
VATS: Video-assisted thoracoscopy.
